# Supplementary material for: Association between proton pump inhibitor use and risk of fracture: A population-based case-control study
Source: PLoS One. 2020 Jul 30;15(7):e0235163. doi: 10.1371/journal.pone.0235163 (PMC7392283; doi:10.1371/journal.pone.0235163)
Supplement: Attachment — Submitted filename: Reviewer_Comments_3rd revision.docx [file pone.0235163.s001.docx]

**Response to reviewer’s comments**

Thanks for reviewer#1's kind comments on our paper. We did reflect the matters you pointed out as much as possible.

1. Study rationale : The authors provided speculations on how the findings would be useful. However, I found “the studies lack information on long-term users. Therefore, we need to reconfirm the influence of the interaction between BP and PPIs on fracture risk in long-term PPI users” not convincing enough. I understand that the authors aimed to “reconfirm” the previous findings. However, they are expected to make the novelties and rationales far clearer and more convincing. More specifically, please clarify how the current findings could contribute to the current science knowledge on top of the previous findings from several meta-analyses (references 18, 19) and a very large nested case-control study (reference 20).

Author’s reply: Thank you for your comments regarding the study novelties. Basically we agreed with you because a novelty is important in academic researches. As we thought our study had the following strengths comparing to previous studies on the same topic. We have added three points separately in the Discussion.

- There were discrepancies in the relationship between the duration of PPI use and fracture risk in the previous meta-analyses, due to applying different definitions of the PPI exposure duration in those studies. Considering the mechanism by which PPI use increases the risk of fracture, our study defined the PPI exposure duration as the cumulative prescription days for 3 years for each subject. With the strength of such research design, we could identify more robust association between PPI use and fracture risk. [Revised manuscript page 12, line 240~247]
- Considering that PPI is the major drug indicated for GERD which affect fractures, this study was conducted only for patients with GERD or gastric ulcer. We enrolled patients with diagnosis of GERD or gastric ulcer, and the association of PPI use and fracture risk was analyzed for the cohort. This approach probably minimized confounding effect related with both gastric problems and fractures [Revised manuscript page 15, line 314~323]
- We applied the same 3-year observation period on PPI exposure before index date (or same date in matching control) to both cases and controls. 3-years is a comparatively long-term observation than the previous studies, and the same length of the observation period could make time-window bias minimize. [Revised manuscript page 15, line 332~337]

2. I agree with what the authors explained about covariates in the analyses. However, I’d like to suggest them to add the explanation they made for weight and BMD into the manuscript, which would make their analyses more convincing.

Author’s reply: Thank you. As per your suggestion, we have added the explanation in the Discussion. [Revised manuscript page 15, line 314~313]

3. Possible time-related biases : I am sorry that my concerns about time-related biases (which was wrongly written as an immortal bias) might have caused a misunderstanding. While I agree with the authors that an immortal bias might not be problematic in their case-control study, I think it is worth discussing further the potential contribution, if any of time-related biases, especially the time-window bias. It is important as the cases and controls were matched by age, osteoporosis diagnosis and Charlson index. It would be an important strength if the time-related biases were already accounted for.

Author’s reply: Thank you for pointing out what we missed. You are right. The same event date and the same observation period were assigned to both cases and controls in our study, which make the possibility of time-window bias minimize. Accordingly, we added this sentences in the Discussion, as the above mentioned in the Answer #1. [Revised manuscript page 15, line 332~337]
